# BMC Endocrine Disorders’ collection of articles on “Reducing inequalities in the Management of Endocrine Disorders”

**DOI:** 10.1186/s12902-022-00998-5

**Published:** 2022-04-11

**Authors:** Alexios-Fotios A. Mentis, George P. Chrousos

**Affiliations:** 1 University Research Institute of Maternal and Child Health and Precision Medicine, National and Kapodistrian University of Athens, Athens, Greece; 2 UNESCO Chair on Adolescent Health Care, National and Kapodistrian University of Athens, Athens, Greece

## Abstract

Endocrine disorders represent a large component of the so-called *“chronic non-communicable diseases”*, which are responsible for the lion share of morbidity and mortality in contemporary societies. As discussed in this retrospective collection of articles, solid evidence from diabetes mellitus, the exemplar of common chronic endocrine disorders, highlights profound inequity in all aspects of endocrine disorders’ management and outcomes that should be considered and addressed at large.

## Main manuscript

*“Leaving no one behind”* and *“Reaching the furthest behind first”* [1] are central axes of the World Health Organization’s agenda for achieving health equity and universal health coverage. Endocrine disorders include several chronic non-communicable diseases - e.g., obesity/metabolic syndrome, diabetes mellitus, among others.- and, hence, represent a major focus of these efforts, as they affect a large proportion of the global population. In this retrospective article collection of *“BMC Endocrine Disorders”,* we assess relevant articles published in the journal earlier, and, using diabetes mellitus as an example, we attempt to examine the apparent inequalities in the management of endocrine disorders.

Disparities in the management of endocrinopathies are prevalent everywhere, from resource-rich to resource-poor settings. Several societal factors, ‑such as low educational background, poverty, aging population, migrant status, certain ethnic/racial backgrounds (e.g., Hispanic/Latino, East Asian, etc.), lack of stable access to food resources, living in non-urban settings, especially coupled with younger age, pediatric background of malnutrition and limited access to electricity, clean water, and/or an organized health care system – all play major negative roles in the *“exemplar”* of endocrinopathies, diabetes mellitus, but also in its precursor forms (prediabetes) and its associated biomarkers (indicatively [2, 3]).

Such societal factors are frequently associated with lack of self-education and self-care practices, – such as proper nutrition and physical activity, and knowledge about diabetes’s management and short‑ and long-term complications and consequences,‑ which, in turn, exacerbate diabetes nosology, especially if the disease is present in an individual for more than 15 years, creating an overall vicious cycle [4]. The negative influence of these factors on the control of diabetes may be mediated through several pathways, including both psychological and physical distress, accentuated by low income levels, decreased access to social support networks, as well as fatalistic approaches towards life [5]. The associations between social and other determinants of health in terms of diabetes control may be quite complex; for instance, patients of a lower socio-economic profile, actively working subjects, and male sex may be associated with lower variation in their dietary patterns and/or exercise [6].

Regarding the treatment of diabetes mellitus, evidence-based approaches, notably systematic reviews and meta-analyses, have confirmed diabetes-related health education’s major role (through institutionally provided training, self-learning or peer education) on glycemic control [7], and the importance of quality of care improvement approaches in reducing diabetes-associated complications in socially disadvantaged populations [8]. Undeniably, several barriers exist, including social and family issues, lack of adequate financial resources, and others (e.g., social security officers) [9]. Nonetheless, sophisticated digital health-based diabetes management tools (e.g.., remote blood glucose monitoring through wearable devices) could address these barriers through an equity lens [10].

In the quest to *“reach the furthest behind first”* as per the World Health Organization’s call-for-action, we hope that the Journal’s readership will perceive this Special Issue as a springboard for both future biomedical research and implementation in clinical practice.

## Data Availability

Not applicable.

## References

[1] Bukhman G, Mocumbi AO, Atun R, Becker AE, Bhutta Z, Binagwaho A (2020). The lancet NCDI poverty commission: bridging a gap in universal health coverage for the poorest billion. Lancet.

[2] Orr CJ, Keyserling TC, Ammerman AS, Berkowitz SA (2019). Diet quality trends among adults with diabetes by socioeconomic status in the US: 1999–2014. BMC Endocr Disord.

[3] Kyrou I, Tsigos C, Mavrogianni C, Cardon G, Van Stappen V, Latomme J (2020). Sociodemographic and lifestyle-related risk factors for identifying vulnerable groups for type 2 diabetes: a narrative review with emphasis on data from Europe. BMC Endocr Disord.

[4] Silva-Tinoco R, Cuatecontzi-Xochitiotzi T, la Torre-Saldaña D, León-García E, Serna-Alvarado J, Orea-Tejeda A (2020). Influence of social determinants, diabetes knowledge, health behaviors, and glycemic control in type 2 diabetes: an analysis from real-world evidence. BMC Endocr Disord.

[5] Walker RJ, Gebregziabher M, Martin-Harris B, Egede LE (2014). Relationship between social determinants of health and processes and outcomes in adults with type 2 diabetes: validation of a conceptual framework. BMC Endocr Disord.

[6] Mutyambizi C, Pavlova M, Hongoro C, Groot W (2020). Inequalities and factors associated with adherence to diabetes self-care practices amongst patients at two public hospitals in Gauteng, South Africa. BMC Endocr Disord.

[7] Ricci-Cabello I, Ruiz-Pérez I, Rojas-García A, Pastor G, Rodríguez-Barranco M, Gonçalves DC (2014). Characteristics and effectiveness of diabetes self-management educational programs targeted to racial/ethnic minority groups: a systematic review, meta-analysis and meta-regression. BMC Endocr Disord.

[8] Terens N, Vecchi S, Bargagli AM, Agabiti N, Mitrova Z, Amato L (2018). Quality improvement strategies at primary care level to reduce inequalities in diabetes care: an equity-oriented systematic review. BMC Endocr Disord.

[9] Davoodi M, Dindamal B, Dargahi H, Faraji-Khiavi F (2022). A phenomenological study on barriers of adherence to medical advice among type 2 diabetic patients. BMC Endocr Disord.

[10] Montero AR, Toro-Tobon D, Gann K, Nassar CM, Youssef GA, Magee MF (2021). Implications of remote monitoring Technology in Optimizing Traditional Self-Monitoring of blood glucose in adults with T2DM in primary care. BMC Endocr Disord.

